# Pervasive environmental contaminant acrolein compromises myocardial geometry and function through the induction of cuproptosis: Acrolein, cuproptosis, and myocardial injury

**DOI:** 10.3724/abbs.2025179

**Published:** 2025-12-18

**Authors:** Xiaohu Wang, Shuyi Zhu, Yipin Zhao, Xinlong Di, Lingfei Cao, Russel J. Reiter, Emily Y. He, Yuan Zhou, Bonglee Kim, Yong Cheng, Jun Ren

**Affiliations:** 1 Department of Cardiology Fuwai Central China Cardiovascular Hospital Henan Provincial People’s Hospital Heart Center Zhengzhou 451464 China; 2 The Cardiovascular Medicine Department The First Affiliated Hospital of Zhengzhou University Zhengzhou 451162 China; 3 Department of Cardiology Zhengzhou University Central China Fuwai Hospital Zhengzhou 451464 China; 4 Department of Cardiology The Second Affiliated Hospital of Zhengzhou University Zhengzhou 450014 China; 5 Henan Key Laboratory of Medical Tissue Regeneration Henan Medical University Xinxiang 453003 China; 6 Department of Cell Systems and Anatomy UT Health San Antonio TX USA; 7 Department of Clinical Immunology Xijing Hospital Air Force Medical University Xi’an 710032 China; 8 Department of Biomedical Informatics School of Basic Medical Sciences Peking University Beijing 100191 China; 9 Department of Pathology College of Korean Medicine Kyung Hee University Hoegidong Dongdaemun-gu Seoul 02447 Republic of Korea; 10 Department of Cardiovascular Medicine The Sixth People’s Hospital of Zhengzhou Zhengzhou 450052 China; 11 Department of Cardiology Zhongshan Hospital Fudan University Shanghai 200032 China; 12 Shanghai Institute of Cardiovascular Diseases Zhongshan Hospital Fudan University Shanghai 200032 China; 13 National Clinical Research Center for Interventional Medicine Shanghai 200032 China

**Keywords:** acrolein, myocardial, remodeling, function, cuproptosis, MTF2

## Abstract

Acrolein, a highly reactive α,β-unsaturated aldehyde found in cigarette smoke, automobile exhaust, industrial emissions, combustion byproducts, cooking and cyclophosphamide chemotherapy, has raised serious health concerns, although the precise mechanism remains unclear. This study is designed to examine the impact of this pervasive environmental pollutant on myocardial geometry and function alongside the underlying cellular mechanisms. Adult C57BL/6 mice are challenged with acrolein (2.5 mg/kg/day, i.p., for 20 days) prior to the evaluation of myocardial geometry and function. Acrolein exposure evokes evident cardiac remodeling (interstitial fibrosis), compromised echocardiographic (enlarged LVESD, compromised ejection fraction and fractional shortening), cardiomyocyte contractile and intracellular Ca ^2+^ capacities [decreased peak shortening, maximal velocity of shortening and relengthening (±dL/dt), and electrically stimulated rise in Fura-2 fluorescence intensity (ΔFFI), prolonged time-to-90% relengthening (TR _90_) and intracellular Ca ^2+^ decay], accompanied by overt mitochondrial damage (ultrastructure, aconitase and mitochondrial protein contents), free radical buildup, apoptosis (Bax, Caspase-3, and Bcl2) and cuproptosis (upregulated SLC31A1, DLAT and FDX1), downregulated the Fe-S cluster proteins ACO2 and NDUFS8 alongside unchanged ATP7A and the ferroptosis markers GPX4 and SLC7A11. The levels of copper-sensing protein metal response element binding transcription factor 2 (MTF2), but not MTF1, are increased by acrolein insult. CB-DOCK2 analysis predicts an interaction between acrolein and the MTF2 dimer within its DNA-binding regions. *In vivo* administration of the cuproptosis inhibitor tetrathiomolybdate (TTM), the mitochondrial antioxidant mitoTEMPO or the nonselective MTF2 inhibitor actinomycin D alleviates acrolein-evoked cardiomyocyte dysfunction (decreased PS, ±dL/dt, and prolonged TR _90_). These findings indicate that acrolein evoked cardiac functional anomalies possibly through MTF2-related control of cuproptosis.

## Introduction

Acrolein is a highly reactive α,β-unsaturated aldehyde and a pervasive environmental contaminant generated through the combustion of organic materials, including fossil fuels, tobacco, and high-temperature cooking of dietary fats, proteins, and carbohydrates [ 1– 3] . It is also endogenously produced during lipid peroxidation and myeloperoxidase-mediated inflammatory responses and can arise as a toxic metabolite of chemotherapeutic agents such as cyclophosphamide [ 4– 6] . Among its environmental sources, cigarette smoke represents a significant contributor to human acrolein exposure [3]. Cigarette smoking remains a major global health concern because of its strong association with a wide range of pathological conditions, including chronic obstructive pulmonary disease, cardiovascular and metabolic disorders, infertility, and cancer [ 7– 9] . A growing body of research has highlighted the role of specific toxicants within cigarette smoke, such as acrolein, in driving genotoxic and mutagenic outcomes, thereby contributing to disease progression and premature mortality [ 9– 14] . Owing to its potent electrophilic nature, acrolein readily forms covalent adducts with nucleophilic residues in proteins, lipids, and DNA, leading to oxidative stress, inflammation, and cellular dysfunction [ 5, 15, 16] . These molecular events underlie its involvement in diverse disease processes, including cardiovascular and neurodegenerative disorders, respiratory illness, and cancer [ 3, 17] . Mechanistically, acrolein induces oxidative stress, endothelial injury, and inflammation, which are hallmark processes implicated in the development of atherosclerosis and myocardial damage [ 1, 3, 6, 16– 19] . Experimental evidence further demonstrated that acrolein impairs cardiomyocyte contractility and promotes maladaptive myocardial remodeling, ultimately contributing to the onset of dilated cardiomyopathy [20]. Given its widespread presence, high reactivity, and pathological potential, acrolein has emerged as an important target for toxicological and biomedical investigations.

Emerging evidence has highlighted a critical role for cuproptosis, a newly characterized, copper-dependent form of regulated cell death, in the pathogenesis of a variety of heart diseases, including pressure overload, heart failure, ischemia‒reperfusion injury and doxorubicin-induced cardiomyopathy [ 21– 24] . This unique process is distinct from other types of cell death, such as apoptosis and ferroptosis, and is driven by copper (Cu ^2+^) accumulation, disruption of mitochondrial function, and aggregation of lipoylated proteins, ultimately resulting in proteotoxic stress and cell death [ 24– 26] . Key regulators of cuproptosis include copper importers [ *e.g.*, solute carrier family 31 member 1 (SLC31A1)], exporters (ATP7A, ATP7B), and copper-binding proteins such as metal-regulatory transcription factors (MTF1, MTF2), all of which collectively maintain cellular Cu ^2+^ homeostasis [25]. Despite its increasing relevance in cardiovascular pathology, the involvement of cuproptosis in cigarette smoking and environmental pollutant-associated cardiac anomalies remains unexplored. Given the prominent role of acrolein as an environmental contaminant originating from fossil fuel combustion, tobacco smoke, and high-temperature cooking of dietary fats, proteins, and carbohydrates, as well as its endogenous generation through cyclophosphamide metabolism [ 1– 6] , its toxic effects on cardiovascular tissues are of growing concern. In parallel, cigarette smoking is known to disrupt systemic trace metal homeostasis, including the perturbation of copper metabolism [ 27, 28] , raising the possibility of a mechanistic connection between smoke exposure and copper-induced cell death.

In this study, we explored the impact of short-term acrolein exposure on myocardial structure and function, with a particular emphasis on mitochondrial damage, oxidative stress, and activation of apoptotic pathways, and we also determined the possible involvement of cuproptosis in acrolein-evoked myocardial injury, aiming to uncover novel mechanistic insights into acrolein-related cardiovascular pathology.

## Materials and Methods

### *In vivo* acrolein exposure and drug treatment

All experimental procedures were approved by the Institutional Animal Care and Use Committee (IACUC) of Zhongshan Hospital Fudan University (Shanghai, China) and the Second Affiliated Hospital of Zhengzhou University (Zhengzhou, China). Adult male C57BL/6 mice obtained from GemPharmatech (Nanjing, China) were administered intraperitoneal injections of acrolein (HY-W012845A, 2.5 mg/kg; MedChemExpress, Monmouth Junction, USA) once daily for 20 consecutive days [ 4, 15, 17] . Animals were housed in a temperature-controlled environment (22 ± 2°C) under a 12-h light/dark cycle with unrestricted access to food and water. To investigate the potential modulatory effects of Cu ^2+^ chelation, mitochondrial protection and MTF2 inhibition on acrolein-induced myocardial alterations, additional cohorts of male C57BL/6 mice received the same acrolein regimen either alone or in combination with the Cu ^2+^ chelator tetrathiomolybdate (5 mg/kg, orally, every 5 days; HY-128530; MedChemExpress) [29], the mitochondrion-targeted antioxidant mitoTEMPO (10 mg/kg, i.p., every 5 days; HY-112879; MedChemExpress) [30], or the broad-spectrum MTF2 inhibitor actinomycin D (0.1 mg/kg, i.p., every 5 days; HY-17559; MedChemExpress) [31]. Following respective drug treatment, cardiomyocyte function was evaluated. Female mice were excluded from this study to reduce the variability associated with sex hormones, which can influence metabolic and cardiovascular parameters.

### Echocardiographic analysis

To assess cardiac function, sedated mice (ketamine at 80 mg/kg and xylazine at 12 mg/kg, i.p.) underwent 2-dimensional guided M-mode echocardiography. Measurements of septal and left ventricular (LV) wall thickness were obtained during both diastole and systole. Fractional shortening (FS) was derived from the following formula: (LVEDD – LVESD) / LVEDD, where LVEDD and LVESD represent the left ventricular end-diastolic and end-systolic diameters, respectively. Additional parameters, including the LV mass, ejection fraction, and heart rate, were averaged from three consecutive cardiac cycles [ 32, 33] .

### Morphological assessment

Following anesthesia (ketamine 80 mg/kg and xylazine 12 mg/kg, i.p.), hearts were removed and tissues were fixed in 10% neutral-buffered formalin at room temperature for 24 h. Samples were then embedded in paraffin and cut into 5-μm-thick sections prior to histological staining, including hematoxylin and eosin (H&E; G1006; Servicebio Technology, Wuhan, China) and Masson’s trichrome (G1005; Servicebio Technology), to determine cardiomyocyte size and interstitial fibrosis. The extent of interstitial fibrosis was quantified by calculating the ratio of the light blue-stained fibrotic area to the entire microscopic field in Masson’s trichrome-stained sections [ 34– 36]

### Isolation of murine cardiomyocytes

Hearts were excised from anesthetized mice (ketamine at 80 mg/kg and xylazine at 12 mg/kg, i.p.) before being mounted onto a home-made Langendorff system maintained at 37°C. Following brief perfusion with Ca ^2+^-free modified Krebs-Henseleit bicarbonate (KHB) buffer containing (135 mM NaCl, 4 mM KCl, 1 mM MgCl _2_,10 mM HEPES, 0.33 mM NaH _2_PO _4_,10 mM glucose, and 10 mM butanedione monoxime, while enzymatic digestion was carried out via Liberase Blendzyme 4 (Hoffmann-La Roche, Indianapolis, USA). Upon completion of digestion, the hearts were detached from the cannula and minced into small fragments to release cardiomyocytes. The extracellular Ca ^2+^ levels were gradually restored to a final concentration of 1.2 mM over a 30-min period. A yield of approximately 70% rod-shaped cardiomyocytes with distinct sarcomere striations was deemed successful [ 33, 35] .

### Cell shortening mechanics

The mechanical properties of cardiomyocytes were evaluated via the SoftEdge MyoCam® system (IonOptix, Westwood, USA).The cells were continuously perfused with a contractile buffer composed of 131 mM NaCl, 4 mM KCl, 1 mM MgCl₂, 1 mM CaCl₂, 10 mM glucose, and 10 mM HEPES and electrically stimulated to induce contraction. Contractile dynamics, including peak shortening (PS), maximal velocities of shortening (+dL/dt) and relengthening (–dL/dt), time-to-peak shortening (TPS), and time-to-90% relengthening (TR _90_), were recorded via IonOptix SoftEdge software [35].

### Intracellular Ca ^2+^ transients

Cardiomyocytes were incubated with 0.5 μM Fura-2/AM to enable intracellular Ca ^2+^ imaging. The fluorescence intensity was quantified via an IonOptix fluorescence photomultiplier tube (PMT) system (IonOptix). Cells were observed under a 40× oil immersion objective and alternately excited at 360 and 380 nm, with the emitted fluorescence collected between 480–520 nm. The Fura-2 fluorescence intensity (FFI) was determined via the ratio of emission from 360 nm to 380 nm excitation. Intracellular Ca ^2+^ clearance was assessed via the decay kinetics of the fluorescence signal [35].

### ROS generation

Intracellular ROS levels were quantified on the basis of the fluorescence generated by the oxidation of the fluorogenic probe 5-(6)-chloromethyl-2′,7′-dichlorodihydrofluorescein diacetate (DCF). Isolated cardiomyocytes were maintained with 10 μM DCF at 37°C for 30 min. Following incubation, the fluorescence intensity was measured using a microplate reader (Synergy 2; Bio-Tek Instruments, Winooski, USA) with excitation and emission wavelengths set at 480 nm and 530 nm, respectively. Nontreated cells lacking fluorescence served as the baseline control to account for background signals [37].

### Determination of mitochondrial ultrastructure and function

Heart tissues were initially fixed in a glutaraldehyde/acrolein solution, dehydrated and embedded in LX112 resin. Ultrathin sections (50 nm) were subsequently stained with and then examined under a H-7000 transmission electron microscope (TEM; Hitachi, Tokyo, Japan) to evaluate the cellular ultrastructure [21]. Mitochondrial function was assessed by measuring the activity of aconitase, a key iron-sulfur enzyme in the citric acid cycle that is highly susceptible to oxidative damage. The assay was performed via an Aconitase-340™ kit (OxisResearch, Portland, USA) [32].

### TUNEL apoptosis assessment

Apoptotic DNA fragmentation was assessed via a TUNEL assay kit (Roche, Indianapolis, USA). Paraffin-embedded tissue sections were treated sequentially with proteinase K (AM2546; ThermoFisher Scientific, Waltham, USA), followed by incubation with terminal deoxynucleotidyl transferase (TdT) and fluorescein-labeled dUTP (Roche). TUNEL-positive nuclei were visualized and quantified under a fluorescence microscope (Olympus, Tokyo, Japan) [21].

### Structure-based drug-protein interaction between acrolein and MTF2

The complex structure of the MTF2 dimer (PDB: 5XFR) and acrolein was predicted by CB-DOCK2 ( https://cadd.labshare.cn/cb-dock2/php/index.php). The results were visualized via the CB-DOCK2 tool [38].

### Immunoblotting

Tissue samples were lysed prior to separation with 7%–12% SDS-PAGE (Mini-PROTEAN II; Bio-Rad, Hercules, USA). Samples were then transferred onto nitrocellulose membranes before incubation with anti-peroxisome proliferator-activated receptor gamma coactivator 1α (PGC1α; ab54481; Abcam, Cambridge, USA), anti-uncoupling protein 2 (UCP2; 14670; CST, Danvers, USA), anti-Bax (2772; CST), anti-Bcl2 (3498; CST), anti-cleaved Caspase-3 (ab32042; Abcam), anti-glutathione peroxidase 4 (GPX4; ab125066; Abcam), anti-solute carrier family 7 member 11 (SLC7A11; ab175186; Abcam), anti-SLC31A1 (CTR1; ab317432; Abcam), anti-ATP7A (ab308524; Abcam), anti-dihydrolipoamide acetyltransferase (DLAT; 12362; CST), anti-ferredoxin 1 (FDX1; ab108257; Abcam), anti-NDUFS8 (ab170936; Abcam), anti-aconitase 2 (ACO2; ab129069; Abcam), anti-MTF1 (ab236401; Abcam), anti-MTF2 (ab314753; Abcam), anti-glyceraldehyde-3-phosphate dehydrogenase (GAPDH; 2118; CST) and anti-α-tubulin (2144; CST) antibodies (all at 1:1000 dilution) at 4°C. Horseradish peroxidase-coupled secondary antibodies (1:5000; #111-035-003; #115-035-003; Jackson ImmunoResearch Labs, West Grove, USA) were added to the membranes. After visualization, immunoblot band density was quantified via a calibrated densitometer (Bio-Rad, Hercules, USA) [ 35, 39] . GAPDH or α-tubulin was used as the loading control.

### Measurement of intracellular Cu ^2+^

Intracellular Cu ^2+^ was quantified via a commercial Cu ^2+^ assay kit (ab272528; Abcam). Myocardial tissues were homogenized and diluted with distilled water, and 0.1 mL aliquots of the resulting mixture were transferred to a 96-well UV-compatible plate to be mixed with detection reagents, followed by thorough mixing. The plate was then incubated at room temperature for 5 min. Fluorescence intensity was measured via the microplate reader at an emission wavelength of 359 nm [21].

### Data analysis

All experimental data are presented as the mean ± SEM. Statistical analysis was performed via Student’s *t* test or one-way analysis of variation (ANOVA) followed by Tukey’s *post hoc* test wherever appropriate. The significance of differences was set at *P*  < 0.05.

## Results

### Pathophysiological influence of acrolein exposure on biometric and echocardiographic parameters

**Figure 1:**
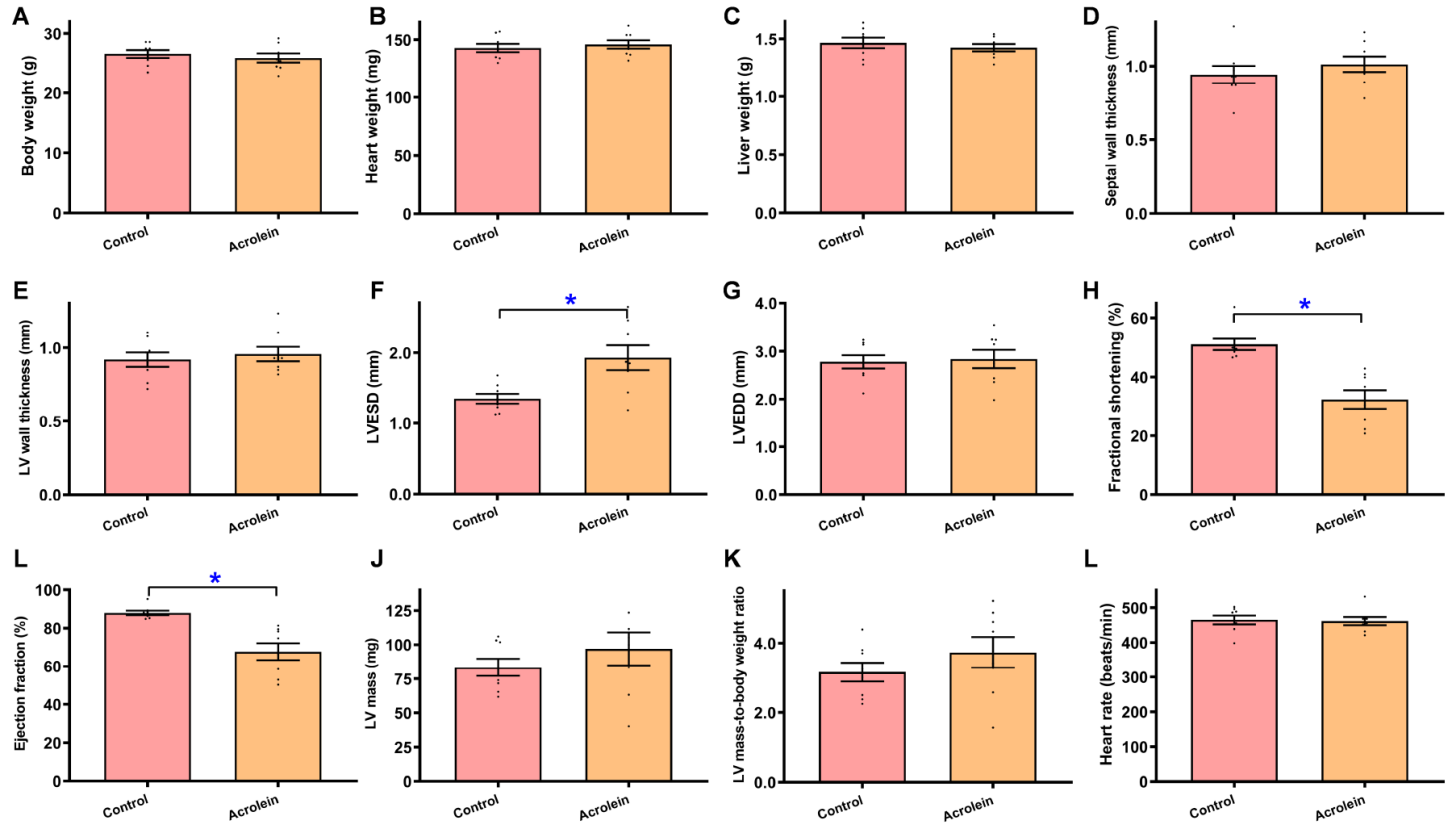
Figure 1 Biometrics and echocardiographic indices of acrolein-challenged mice Mice were exposed to acrolein at 2.5 mg/kg/day (i.p.) for 20 days. (A) Body weight. (B) Heart weight. (C) Liver weight. (D) Septal thickness. (E) LV posterior wall thickness. (F) LV end systolic diameter (LVESD). (G) LV end diastolic diameter (LVEDD). (H) Fractional shortening. (I) Ejection fraction. (J) LV mass. (K) Normalized LV mass (to body weight). (L) Heart rate. Data are presented as the mean ± SEM, n = 8 mice per group, *P < 0.05 between the indicated groups.

For the purpose of examining the impact of acrolein on myocardial geometry and function, we conducted short-term *in vivo* exposure of acrolein in mice. Acrolein exposure did not affect body or organ (heart and liver) weights ( Figure 1A–C). An evaluation of myocardial geometry and function via echocardiography revealed that acrolein exposure overtly increased LV ESD and suppressed fractional shortening and ejection fraction without affecting septal and LV wall thickness, LV EDD, LV mass (absolute or normalized) and heart rate ( Figure 1D–L). These data suggest that short-term exposure to low-dose acrolein compromised myocardial geometry and function without affecting body or organ weights in mice.

### Pathophysiological influence of acrolein exposure on contractile and intracellular Ca ^2+^ properties of cardiomyocytes

**Figure 2:**
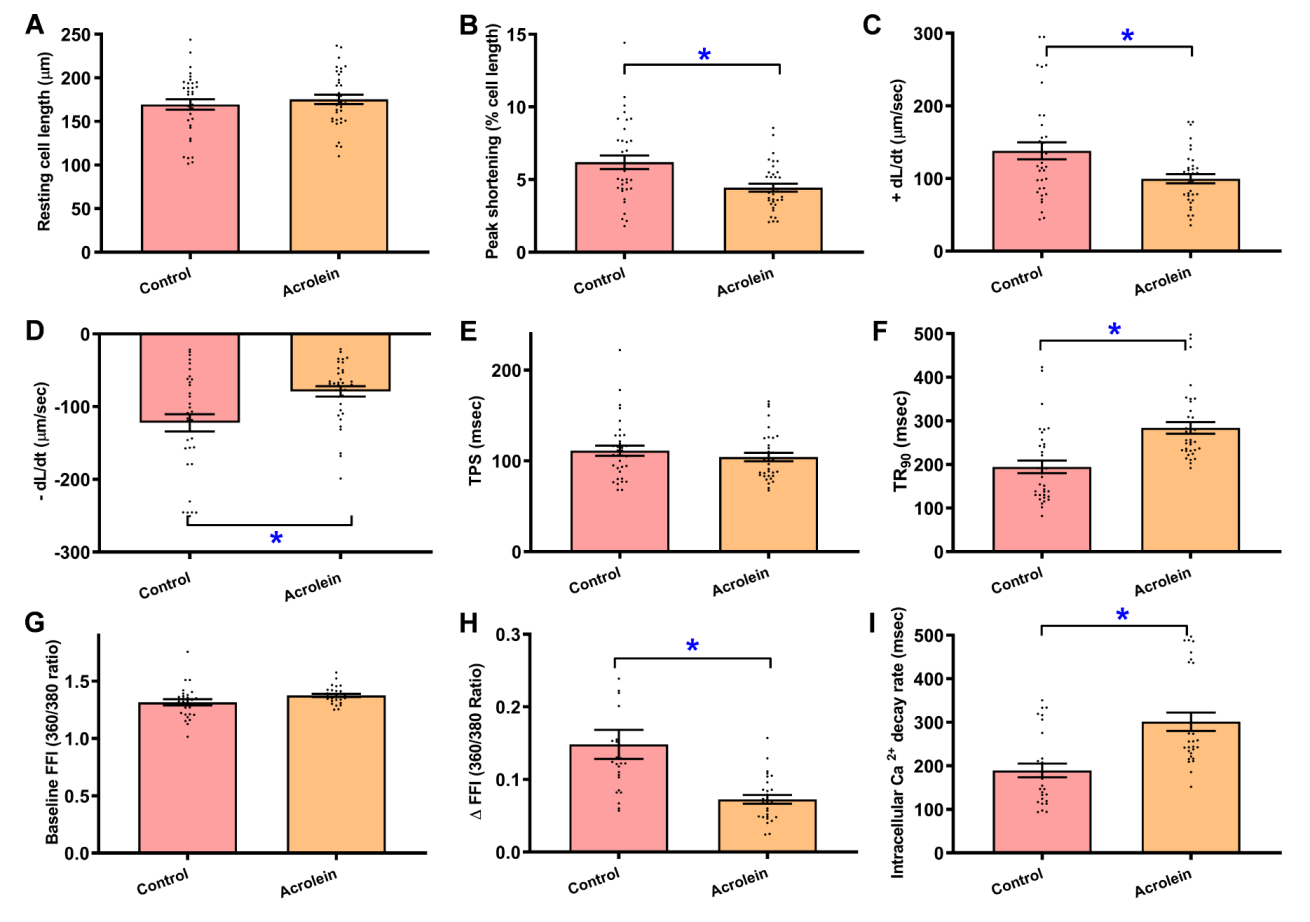
Figure 2 Cardiomyocyte contractile function and intracellular Ca ^2+^ properties in acrolein-challenged mice Mice were exposed to acrolein at 2.5 mg/kg/day (i.p.) for 20 days. (A) Resting cell length. (B) Peak shortening (% of resting cell length). (C) Maximal velocity of shortening (+ dL/dt). (D) Maximal velocity of relengthening (–dL/dt). (E) Time-to-peak shortening (TPS). (F) Time-to-90% relengthening (TR90). (G) Resting Fura-2 fluorescence intensity (FFI). (H) Electrically stimulated rise in FFI (ΔFFI). (I) Intracellular Ca2+ clearance rate. Mean ± SEM, n = 35 cells (panel A–F) or 28 cells (panel G–I) from 3 mice per group, * P < 0.05 between the indicated groups.

Further assessment of cardiomyocyte function revealed that acrolein exposure significantly decreased the maximal velocity of shortening/relengthening (±dL/dt) and peak shortening (PS) capacity, increased the time to 90% relengthening (TR _90_) and unchanged the resting cell length and time to PS (TPS) ( Figure 2A–F). To decipher the possible scenario(s) underlying acrolein exposure-evoked myocardial dysfunction, intracellular Ca ^2+^ handling was assessed via Fura-2 fluorescence. Our data shown in Figure 2G–I revealed that acrolein exposure significantly compromised the electrically-stimulated increase in intracellular Ca ^2+^ (ΔFFI) and delayed intracellular Ca ^2+^ clearance, with little change in the resting intracellular Ca ^2+^. These data suggest that short-term exposure to low-dose acrolein disturbed the mechanical function and intracellular Ca ^2+^ handling of cardiomyocytes.

### Pathophysiological impact of acrolein on myocardial histology and ROS accumulation

**Figure 3:**
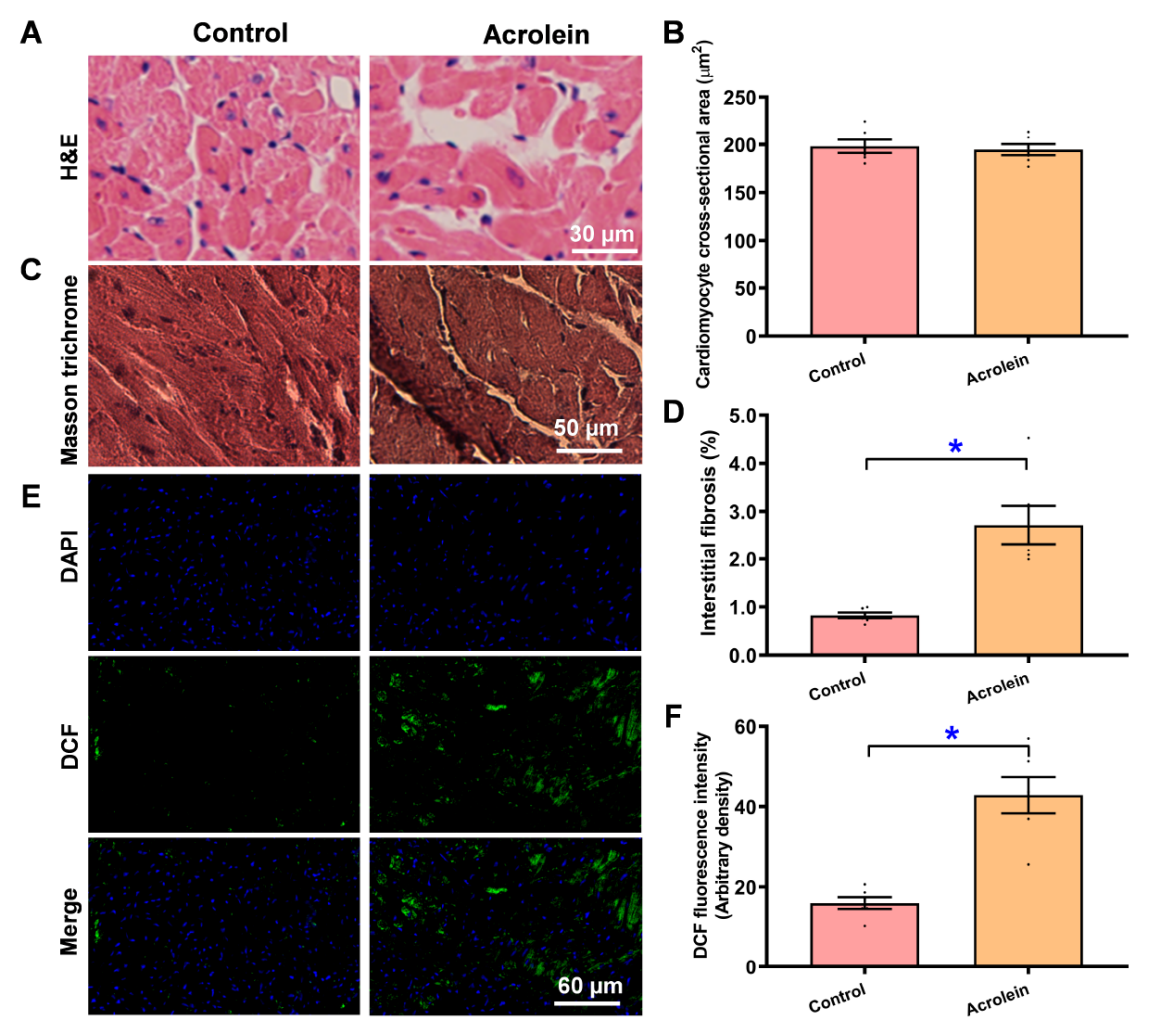
Figure 3 H&E and Masson’s trichrome staining and free radical accumulation in the myocardium of acrolein-challenged mice Mice were exposed to acrolein at 2.5 mg/kg/day (i.p.) for 20 days. (A) Representative micrographs from H&E staining. (B) Pooled cardiomyocyte cross-sectional area. (C) Representative Masson trichrome staining. (D) Pooled interstitial fibrosis analysis. (E) Representative DCF staining (DAPI) top row, DCF) middle row and merged) bottom row). (F) Pooled DCF fluorescence. Data are presented as the mean ± SEM, n = 6 mice per group, *P < 0.05 between the indicated groups.

To evaluate the impact of acrolein exposure on myocardial morphology and oxidative stress, H&E, Masson’s trichrome, and DCF staining were conducted. Our data shown in Figure 3A–F indicate evident interstitial fibrosis and ROS accumulation in murine hearts following short-term acrolein exposure. The cardiomyocyte cross-sectional area was unaffected by short-term acrolein exposure, which was in line with the unchanged heart weight and LV mass. These data suggest that short-term exposure to low-dose acrolein prompts myocardial interstitial fibrosis and free radical damage in the absence of altered cardiomyocyte size.

### Pathophysiological influence of acrolein on the mitochondrial ultrastructure, mitochondrial function, and apoptosis

**Figure 4:**
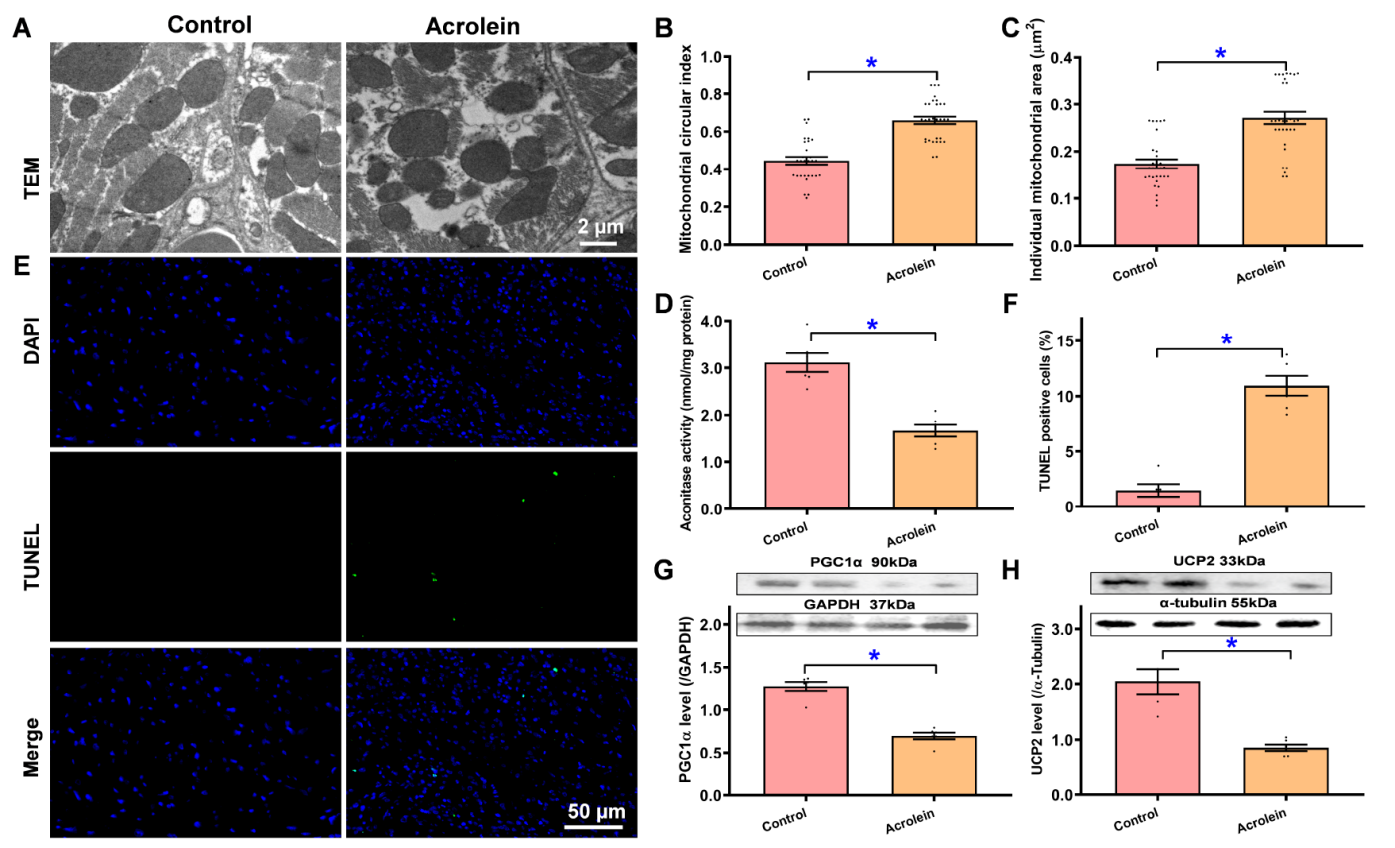
Figure 4 TEM ultrastructure, mitochondrial function, and TUNEL apoptosis in the myocardium of acrolein-challenged mice Mice were exposed to acrolein at 2.5 mg/kg/day (i.p.) for 20 days. (A) Representative TEM ultrastructure for damaged and/or deformed mitochondria. (B) Mitochondrial circular index (short-to-long axis ratio). (C) Mitochondrial area (individual). (D) Mitochondrial function assessed via aconitase activity assay. (E) Representative TUNEL staining depicting apoptosis (DAPI) top row. (TUNEL) middle row. (bottom) merged). (F) Pooled TUNEL staining depicting the percentage of TUNEL-positive apoptotic cells. (G) Level of the mitochondrial protein PGC1α. (H) Level of the mitochondrial protein UCP2. Inset: Representative gel blots showing PGC1α and UCP2 using specific antibodies (GAPDH or α-tubulin as loading controls). Data are presented as the mean ± SEM, n = 30–31 mitochondria from 5 micrographs (panels B,C) or 6 mice (all other panels) per group, *P < 0.05 between the indicated groups.

Evaluation of the TEM ultrastructure revealed that acrolein evoked pronounced alterations in the myocardial ultrastructure, manifested as mitochondrial swelling (increased mitochondrial circularity and size) and myofibrillar disarray ( Figure 4A–C). This is supported by the loss of mitochondrial aconitase activity (indicating mitochondrial injury) ( Figure 4D). Assessment of TUNEL apoptosis indicated that short-term acrolein exposure evoked evident myocardial apoptosis, along with downregulation of the mitochondrial proteins PGC1α and UCP2 ( Figure 4E–H). These findings indicate that short-term exposure to low-dose acrolein evoked pathological changes in the mitochondrial ultrastructure, function and integrity and provoked apoptotic cell death.

### Pathophysiological influence of acrolein exposure on apoptotic, ferroptotic, Cu ^2+^ regulatory, and Fe-S cluster proteins

**Figure 5:**
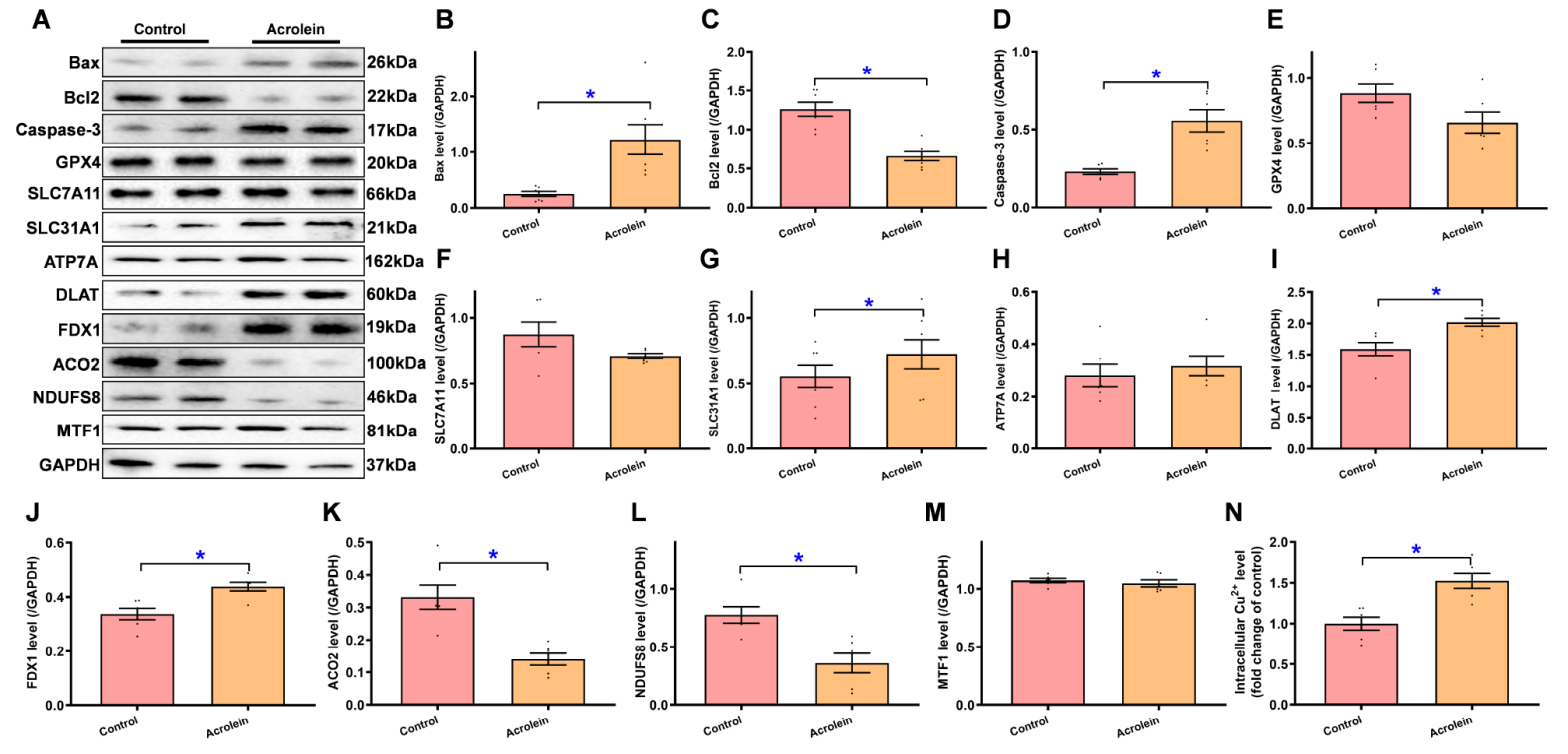
Figure 5 Levels of protein markers for apoptosis, ferroptosis, cuproptosis, and mitochondrial complexes as well as intracellular Cu ^2+^ in hearts from acrolein-challenged mice Mice were exposed to acrolein at 2.5 mg/kg/day (i.p.) for 20 days. (A) Representative immunoblots depicting the levels of Bax, Bcl2, caspase-3, GPX4, SLC7A11, SLC31A1, ATP7A, DLAT, FDX1, ACO2, NDUFS8 and MTF1 using specific antibodies (GAPDH as a loading control). (B) Level of Bax. (C) Level of Bcl2. (D) Level of Caspase-3. (E) Level of GPX4. (F) Level of SLC7A11. (G) Level of SLC31A1 (CTR1). (H) Level of ATP7A. (I) Level of DLAT. (J) Level of FDX1. (K) Level of ACO2. (L) Level of NDUFS8. (M) Level of MTF1. (N) Intracellular Cu2+ level. Data are presented as the mean ± SEM. n = 6–7 hearts per group. * P < 0.05 between the indicated groups.

Considering the reported involvement of oxidative stress and apoptosis in acrolein-induced cell and tissue damage [ 2, 4, 5, 16] , the effects of short-term acrolein exposure on protein markers of oxidative stress, apoptosis, and ferroptosis, with a special focus on Cu ^2+^ regulatory and Fe-S cluster mitochondrial complex proteins, were investigated. Western blotting results clearly revealed the upregulation of the proapoptotic proteins Bax and Caspase-3 (cleaved) and the downregulation of the antiapoptotic protein Bcl-2 following acrolein challenge ( Figure 5A–D). Although acrolein exposure failed to provoke any notable changes in the ferroptosis markers GPX4 and SLC7A11 ( Figure 5A,E,F), the pervasive environmental contaminant significantly increased the levels of the Cu ^2+^ importers SLC31A1 (CTR1), DLAT and FDX1 while downregulating the Fe-S cluster mitochondrial complex proteins ACO2 and NDUFS8 without affecting the Cu ^2+^ exporter ATP7A and the Cu ^2+^-sensing protein MTF1 ( Figure 5A,G–M). Finally, acrolein exposure overtly increased intracellular Cu ^2+^ level within the murine myocardium ( Figure 5N). These findings suggest that exposure to low-dose acrolein evoked the accumulation of intracellular Cu ^2+^, apoptosis, and cuproptosis, with subtle changes in ferroptotic protein markers.

### Interaction between acrolein and the Cu ^2+^-sensing molecule MTF2

**Figure 6:**
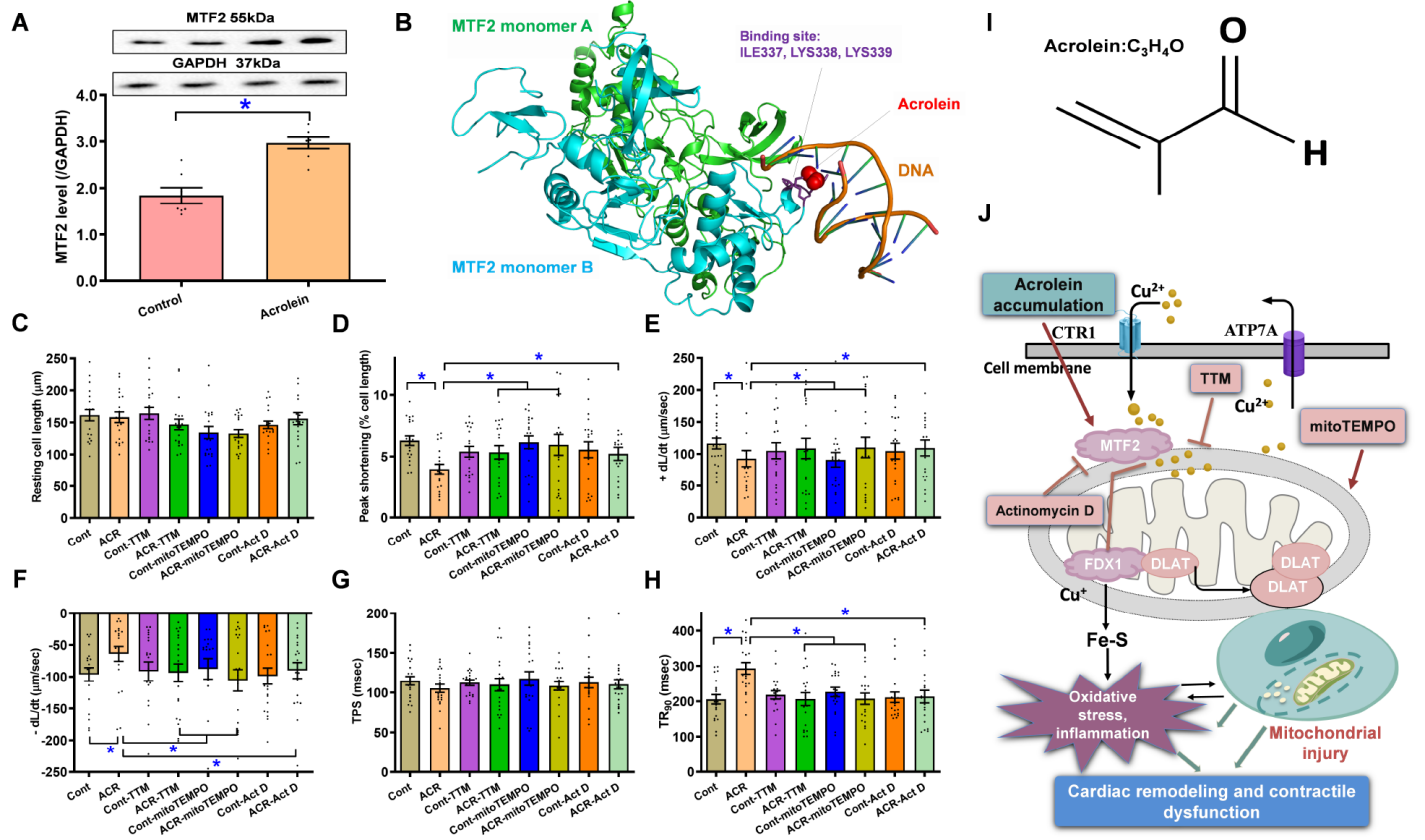
Figure 6 Levels of MTF2, interaction between acrolein and MTF2, and impact of TTM, mitoTEMPO, or Act D on acrolein **-**evoked cardiomyocyte contractile defects Adult C57BL/6 mice were injected with acrolein (2.5 mg/kg/day, i.p.) for 20 days in the presence or absence of TTM (5 mg/kg, p.o., every 5 days), mitoTEMPO (10 mg/kg, i.p., every 5 days) or Act D (0.1 mg/kg, i.p., every 5 days) prior to assessment of mechanical function. (A) Level of MTF2 following acrolein challenge (2.5 mg/kg/day, i.p.). (B) Interaction between acrolein and the MTF2 dimer predicted by CB-DOCK2. (C–H) Impact of TTM and actinomycin D on acrolein-induced cardiomyocyte dysfunction) c) Resting cell length. (D) Peak shortening. (E) +dL/dt. (F) –dL/dt. (G) Time-to-peak shortening (TPS). (H) Time-to-90% lengthening (TR90). (I) Chemical structure of acrolein. (J) Schematic diagram illustrating acrolein-induced myocardial responsiveness (including Cu2+ regulatory and Fe-S cluster proteins, mitochondrial integrity, cell death, myocardial remodeling and function). Data are presented as the mean ± SEM, n = 7 mice (in panel A) or 20 cells from 3 mice (in panel C–H) per group. *P < 0.05 between the indicated groups.

Next, we evaluated the levels of Cu ^2+^ sensing protein MTF2 and found that the levels of MTF2 were markedly elevated in response to short-term acrolein challenge ( Figure 6A). To discern the possible mechanism(s) underlying the acrolein exposure-evoked activation of cuproptosis, the potential interaction between acrolein and the essential Cu ^2+^ sensing molecule MTF2 was assessed. The complex structure of the MTF2 dimer (PDB: 5XFR) and acrolein was predicted via CB-DOCK2 software ( https://cadd.labshare.cn/cb-dock2/php/index.php). Our results indicate that acrolein interacts with the DNA-binding regions of MTF2 (docking score = –1.7), thus plausibly interfering with the DNA-binding activity of the transcription factor MTF2 ( Figure 6B). These findings suggest a possible direct interaction between the acrolein molecule and the Cu ^2+^ sensing protein MTF2.

### Protective effect of tetrathiomolybdate (TTM), mitoTEMPO, and actinomycin D on acrolein-evoked changes in cardiomyocyte function

To investigate the involvement of cuproptosis, mitochondria and Cu ^2+^ sensing molecule MTF2 in acrolein-elicited myocardial anomalies, mice were challenged with acrolein (2.5 mg/kg/day, i.p., for 20 days) in the absence or presence of the cooper chelator TTM, the mitochondrial antioxidant mitoTEMPO, or the Cu ^2+^ sensing MTF2 inhibitor actinomycin D prior to determining cardiomyocyte function. None of the pharmacological inhibitors had any effect on the contractile parameters of cardiomyocytes themselves. However, acrolein-instigated cardiomyocyte anomalies (dropped ±dL/dt and PS alongside prolonged TR _90_ and unchanged resting cell length or TPS) were effectively mitigated by TTM, mitoTEMPO or actinomycin D ( Figure 6C–H). These data favor a likely role for cuproptosis, mitochondrial integrity and Cu ^2+^ sensing in acrolein-instigated myocardial geometric and contractile responsiveness.

## Discussion

Our current study revealed that *in vivo* exposure to acrolein, a toxic α,β-unsaturated aldehyde prevalent in the combustion of organic materials such as fossil fuels, tobacco, and the high-temperature cooking of dietary fats, proteins, and carbohydrates [ 1– 3, 6, 40] , as well as a byproduct of cyclophosphamide metabolism [ 4, 5] , induces marked alterations in cardiac geometry and function. These pathological changes are strongly associated with mitochondrial injury and the activation of cuproptosis, a copper-dependent form of regulated cell death [25]. Importantly, pharmacological inhibition of cuproptosis and the mitochondrion-targeted antioxidant mitoTEMPO effectively attenuated acrolein-induced cardiomyocyte injury, suggesting a potentially targetable mechanism in acrolein-related cardiac dysfunction. At the mechanistic level, short-term acrolein exposure led to significant upregulation of MTF2, a copper-responsive transcription factor involved in maintaining intracellular copper homeostasis [41]. This finding implicates the MTF2 molecule as a potential molecular link between environmental aldehyde exposure and copper-induced cytotoxicity. Together, these findings provide novel insights into the cardiotoxic effects of acrolein and highlight the involvement of the cuproptosis-dependent pathway in pervasive environmental contaminant-induced myocardial remodeling and dysfunction. Targeting this pathway may represent a promising therapeutic strategy for preventing cardiovascular injury in individuals exposed to acrolein from cigarette smoke or other environmental sources.

Acrolein is a highly reactive aldehyde that contributes to tissue injury across multiple organ systems. In the respiratory tract, it has been implicated in the development of asthma, acute lung injury, and potentially lung cancer through its interaction with tumor suppressor DNA and promotion of p53 mutations [ 3, 17, 19, 40] . Notably, sustained acrolein exposure has also been associated with neurodegenerative pathology, including features characteristic of Alzheimer’s disease, suggesting a potential mechanistic link between cigarette smoking and an increased incidence of neurodegenerative disorders [ 17, 42, 43] . Owing to its strong electrophilicity, acrolein readily reacts with components of respiratory lining fluids and cellular macromolecules, thereby disrupting critical biological processes such as inflammation, mucociliary clearance, gene regulation, and alveolar‒capillary barrier integrity [ 16, 40] . It also alters the expression of genes involved in surface receptor signaling, transcriptional regulation, and the ubiquitin‒proteasome system [ 16, 40] . In our current study, *in vivo* acrolein exposure led to pronounced alterations in myocardial geometry, as evidenced by increased left ventricular end-systolic diameter (LVESD) and interstitial fibrosis and a reduction in ejection fraction, fractional shortening, and cardiomyocyte contractility. These impairments were accompanied by disturbed intracellular Ca ^2+^ handling, as evidenced by diminished ΔFFI and delayed Ca²⁺ clearance. Our findings are in line with previous reports demonstrating that chronic oral acrolein exposure results in left ventricular chamber dilation, systolic dysfunction, and impaired myocardial relaxation, hallmark features of dilated cardiomyopathy [20]. Importantly, our results suggest that these deleterious effects are due primarily to direct cardiomyocyte injury rather than secondary alterations in the extracellular microenvironment, such as endothelial cells, fibroblasts, or intercellular junctions [ 14, 44– 46] . Furthermore, these pathological changes were associated with increased ROS production and increased apoptosis but not ferroptosis, supporting a mechanistic link between oxidative stress, apoptotic signaling, and acrolein-induced myocardial dysfunction.

Multiple mechanistic pathways may contribute to the myocardial injury observed following acrolein exposure. First, acrolein is known to impair mitochondrial integrity, increase ROS production, and trigger apoptosis, features that align with key hallmarks of cuproptosis, a Cu ^2+^-dependent form of regulated cell death [25]. In our study, acrolein exposure led to significant changes in protein markers associated with cuproptosis and Fe-S cluster metabolism, including elevated intracellular Cu ^2+^, SLC31A1, DLAT, and FDX1, as well as downregulated ACO2 and NDUFS8. Pharmacological inhibition of cuproptosis via TTM markedly alleviated acrolein-induced cardiomyocyte dysfunction, indicating an essential role for cuproptosis in the observed pathology. Consistent with the findings of mitochondrial impairment, we noted reduced aconitase activity and mitochondrial ultrastructural injury following acrolein exposure, suggesting compromised oxidative phosphorylation and ATP synthesis. Acrolein also enhanced ROS production, likely exacerbating oxidative stress, membrane depolarization, and downstream inflammatory signaling. This finding is further supported by the beneficial effect of mitoTEMPO against acrolein-induced cardiotoxicity, which is in line with the ability of the administration of naturally occurring antioxidants to protect mitochondria [ 19, 47, 48] . Furthermore, our data suggest that acrolein may directly modulate intracellular Cu ^2+^ metabolism via interaction with the Cu ^2+^-sensing transcription factor MTF2, potentially increasing Cu ^2+^ import through SLC31A1. This hypothesis is supported by the protective effects of actinomycin D, a broad-spectrum transcriptional inhibitor known to suppress MTF2 activity, in acrolein-challenged murine cardiomyocytes. Interestingly, this effect occurred without detectable changes in the Cu ^2+^ exporter ATP7A, indicating a net increase in intracellular Cu ^2+^ accumulation—an event that may potentiate cuproptosis onset ( Figure 6I,J).

In addition to promoting cuproptosis and oxidative stress, acrolein disrupted intracellular Ca ^2+^ handling, as evidenced by decreased ΔFFI and delayed Ca²⁺ clearance. Given the known vulnerability of Ca ^2+^ regulatory mechanisms to oxidative stress [49], this disturbance is likely a secondary consequence of ROS accumulation and mitochondrial injury. Taken together, these findings point to a multifactorial mechanism of acrolein-evoked myocardial dysfunction driven by mitochondrial damage, oxidative stress, dysregulated copper homeostasis, and impaired Ca ^2+^ signaling. These interconnected pathways may offer therapeutic targets for preventing or mitigating cardiovascular injury associated with environmental acrolein exposure.

Nevertheless, this study has several limitations. First and foremost, apoptosis, ferroptosis, and other forms of cell death or cell injury, such as endoplasmic reticulum stress and DNA damage, share overlapping molecular features with cuproptosis [ 50, 51] . Although our data revealed changes in proteins associated with lipoylated protein aggregation, degradation of Fe‒S cluster proteins and mitochondrial stress along with subtle alterations in ferroptosis and increased apoptosis, we did not systematically rule out the involvement of other cell death or cell stress pathways. At this stage, fully disentangling cuproptosis from other types of cell death is rather difficult. In particular, cuproptosis is often accompanied by apoptosis, as observed in our study. Thus, caution must be taken in the indication of cuproptosis involvement but not exclusive to other forms of cell death in the face of acrolein exposure. Second, the heart is composed of multiple cell types, including endothelial cells, fibroblasts, and resident and infiltrating immune cells, all of which contribute to acrolein cardiotoxicity [52]. While our current study focused mainly on myocardial tissues and inferred cardiomyocyte-intrinsic effects on myocardial function and mitochondrial impairment, potential contributions from noncardiomyocytes cannot be excluded. For example, endothelial cells are highly sensitive to oxidative stress. Fibroblasts may contribute to remodeling, fibrosis, or proinflammatory cytokine release in response to aldehyde-induced stress, whereas immune cells, such as macrophages, may infiltrate myocardial tissues in response to acrolein and amplify inflammatory signaling and tissue injury. Third, actinomycin D is a broad-spectrum transcriptional inhibitor, and its biological effects cannot be attributed exclusively to the inhibition of MTF2. In our hands, actinomycin D was used as a tool to assess the transcriptional dependence of the observed phenotype, although its use does not constitute direct evidence of MTF2-specific involvement. Future studies employing more targeted approaches, such as siRNA-mediated knockdown, CRISPR interference, or MTF2-specific inhibitors (unavailable currently), are warranted to precisely delineate the role of MTF2 in the context of acrolein-induced cardiotoxicity. Finally, a single dose of acrolein was used in our study, largely because low-dose acrolein (1–5 mg/kg, i.p.) had a minimal effect on animal survival and organ weight compared with high-dose acrolein (10–20 mg/kg) [4]. A full-spectrum dose‒response analysis would strengthen the toxicological profile of pervasive environmental contaminants.

Cuproptosis, a recently identified form of regulated cell death driven by intracellular Cu ^2+^ accumulation and mitochondrial dysfunction [25], has emerged as a novel contributor to cardiac pathology. The findings from our study indicate that exposure to the pervasive environmental pollutant acrolein induces adverse myocardial remodeling and functional decline. These pathological changes are closely associated with mitochondrial damage and the activation of cuproptosis. Our findings support the hypothesis that cuproptosis plays a central role in mediating the cardiotoxic effects of acrolein and, by extension, cigarette smoke. However, while these results provide new insights into the mechanism underlying acrolein-induced cardiovascular damage, the specific contribution of cuproptosis relative to other cell death pathways and the roles of additional reactive α,β-unsaturated aldehydes remain to be fully elucidated. Further investigations are needed to clarify the molecular interplay between acrolein exposure, copper homeostasis, and broader signaling networks within the myocardium.

## References

[1] Hikisz P, Jacenik D (2023). Diet as a source of acrolein: molecular basis of aldehyde biological activity in diabetes and digestive system diseases. Int J Mol Sci.

[2] Scianò F, Bernardoni BL, D′Agostino I, Ferrara G, Tafi A, Garavaglia S, La Motta C (2025). Toxic aldehydes in cooking vegetable oils: generation, toxicity and disposal methods. Food Chem X.

[3] Hikisz P, Jacenik D (2023). The tobacco smoke component, acrolein, as a major culprit in lung diseases and respiratory cancers: molecular mechanisms of acrolein cytotoxic activity. Cells.

[4] Conklin DJ, Haberzettl P, Jagatheesan G, Baba S, Merchant ML, Prough RA, Williams JD (2015). Glutathione s-transferase P protects against cyclophosphamide-induced cardiotoxicity in mice. Toxicol Appl Pharmacol.

[5] Alizadehasl A, Shahrami B, Rahbarghazi R, Yalameh Aliabadi A, Hosseini Jebelli SF, Afsari Zonooz Y, Hakimian H (2024). Post-transplant cyclophosphamide-induced cardiotoxicity: a comprehensive review. J Cardiovasc Thorac Res.

[6] Šebela M, Rašková M (2023). Polyamine-derived aminoaldehydes and acrolein: cytotoxicity, reactivity and analysis of the induced protein modifications. Molecules.

[7] Schiliro M, Vogel ER, Paolini L, Pabelick CM (2021). Cigarette smoke exposure, pediatric lung disease, and COVID-19. Front Physiol.

[8] Konstantinou E, Fotopoulou F, Drosos A, Dimakopoulou N, Zagoriti Z, Niarchos A, Makrynioti D (2018). Tobacco-specific nitrosamines: a literature review. Food Chem Toxicol.

[9] Tweed JO, Hsia SH, Lutfy K, Friedman TC (2012). The endocrine effects of nicotine and cigarette smoke. Trends Endocrinol Metab.

[10] Ishida M, Sakai C, Kobayashi Y, Ishida T (2024). Cigarette smoking and atherosclerotic cardiovascular disease. J Atheroscler Thromb.

[11] Mallah MA, Soomro T, Ali M, Noreen S, Khatoon N, Kafle A, Feng F (2023). Cigarette smoking and air pollution exposure and their effects on cardiovascular diseases. Front Public Health.

[12] Gaalema DE, Khadanga S, Pack QR (2023). Clinical challenges facing patient participation in cardiac rehabilitation: cigarette smoking. Expert Rev Cardiovasc Ther.

[13] Liu F, Liu Y, Zhuang Z, Ma J, Xu X, Zhang W, Peng H (2020). Beclin1 haploinsufficiency accentuates second-hand smoke exposure-induced myocardial remodeling and contractile dysfunction through a STING-mediated mechanism. J Mol Cell Cardiol.

[14] Wang S, Chen X, Zeng B, Xu X, Chen H, Zhao P, Hilaire ML (2020). Knockout of macrophage migration inhibitory factor accentuates side-stream smoke exposure-induced myocardial contractile dysfunction through dysregulated mitophagy. Pharmacol Res.

[15] Wang CC, Chen HJ, Chan DC, Chiu CY, Liu SH, Lan KC (2021). Low-dose acrolein, an endogenous and exogenous toxic molecule, inhibits glucose transport via an inhibition of Akt-regulated GLUT4 signaling in skeletal muscle cells. Int J Mol Sci.

[16] Moghe A, Ghare S, Lamoreau B, Mohammad M, Barve S, McClain C, Joshi-Barve S (2015). Molecular mechanisms of acrolein toxicity: relevance to human disease. Toxicol Sci.

[17] Huang YJ, Jin MH, Pi RB, Zhang JJ, Ouyang Y, Chao XJ, Chen MH (2013). Acrolein induces Alzheimer’s disease-like pathologies in vitro and in vivo. Toxicol Lett.

[18] Kim M, Han CH, Lee MY (2014). NADPH oxidase and the cardiovascular toxicity associated with smoking. Toxicol Res.

[19] Aydın B, Oğuz A, Şekeroğlu V, Atlı Şekeroğlu Z (2022). Whey protein protects liver mitochondrial function against oxidative stress in rats exposed to acrolein. Arch Industrial Hyg Toxicol.

[20] Ismahil MA, Hamid T, Haberzettl P, Gu Y, Chandrasekar B, Srivastava S, Bhatnagar A (2011). Chronic oral exposure to the aldehyde pollutant acrolein induces dilated cardiomyopathy. Am J Physiol Heart Circulatory Physiol.

[21] Liu Y, Shen M, Zhu S, Du Z, Lin L, Ma J, Reiter RJ (2025). Metallothionein rescues doxorubicin cardiomyopathy via mitigation of cuproptosis. Life Sci.

[22] Kong B, Zheng X, Hu Y, Zhao Y, Hai J, Ti Y, Bu P (2025). Sirtuin3 attenuates pressure overload-induced pathological myocardial remodeling by inhibiting cardiomyocyte cuproptosis. Pharmacol Res.

[23] Li ZZ, Guo L, An YL, Yu WJ, Shi DY, Lin QY, Zhang B (2025). Evolocumab attenuates myocardial ischemia/reperfusion injury by blocking PCSK9/LIAS-mediated cuproptosis of cardiomyocytes. Basic Res Cardiol.

[24] Liu Z, Gan Y, Shen Z, Cai S, Wang X, Li Y, Li X (2024). Role of copper homeostasis and cuproptosis in heart failure pathogenesis: implications for therapeutic strategies. Front Pharmacol.

[25] Yang L, Yang P, Lip GYH, Ren J (2023). Copper homeostasis and cuproptosis in cardiovascular disease therapeutics. Trends Pharmacol Sci.

[26] Xu H, Yu Z, Zhu J, Liu H, Chen X, Jiang J, Zhu M (2024). Types of cell death in diabetic cardiomyopathy: insights from animal models. Acta Biochim Biophys Sin.

[27] Afridi HI, Kazi TG, Talpur FN, Brabazon D (2015). Evaluation of trace and toxic elements in the samples of different cigarettes and their impact on human health of irish diabetes mellitus patients. Clin Lab.

[28] Nakhaee S, Amirabadizadeh A, Ataei M, Ataei H, Zardast M, Shariatmadari MR, Mousavi-Mirzaei SM (2021). Comparison of serum concentrations of essential and toxic elements between cigarette smokers and non-smokers. Environ Sci Pollut Res.

[29] Kim P, Zhang CC, Thoröe-Boveleth S, Buhl EM, Weiskirchen S, Stremmel W, Merle U (2021). Analyzing the therapeutic efficacy of bis-choline-tetrathiomolybdate in the Atp7b–/– copper overload mouse model. Biomedicines.

[30] Liu SQ, Xie SY, Zhang T, Zhang H, Chen MY, Xing Y, Zhao N (2024). Impeding nucleotide-binding oligomerization domain-like receptor 3 inflammasome ameliorates cardiac remodeling and dysfunction in obesity-associated cardiomyopathy. J Am Heart Assoc.

[31] Merkel O, Wacht N, Sifft E, Melchardt T, Hamacher F, Kocher T, Denk U (2012). Actinomycin D induces p53-independent cell death and prolongs survival in high-risk chronic lymphocytic leukemia. Leukemia.

[32] Wang X, Li FJ, Cheng Y, Chen S, Zhu S, Zhang Y, Reiter RJ (2025). Activation of protein kinase B rescues against thapsigargin-elicited cardiac dysfunction through regulation of NADPH oxidase and ferroptosis. Chem-Biol Interact.

[33] Yuan M, Ceylan AF, Gao R, Zhu H, Zhang Y, Ren J (2023). Selective inhibition of the NLRP3 inflammasome protects against acute ethanol-induced cardiotoxicity in an FBXL2-dependent manner. Acta Biochim Biophys Sin.

[34] Lu Q, Qin X, Chen C, Yu W, Lin J, Liu X, Guo R (2024). Elevated levels of alcohol dehydrogenase aggravate ethanol-evoked cardiac remodeling and contractile anomalies through FKBP5-yap-mediated regulation of ferroptosis and ER stress. Life Sci.

[35] Chen Y, Zhao J, Ye H, Ceylan-Isik AF, Zhang B, Liu Q, Yang Y (2024). Beneficial impact of cardiac heavy metal scavenger metallothionein in sepsis-provoked cardiac anomalies dependent upon regulation of endoplasmic reticulum stress and ferroptosis but not autophagy. Life Sci.

[36] Yu W, Xu H, Sun Z, Du Y, Sun S, Abudureyimu M, Zhang M (2023). TBC1D15 deficiency protects against doxorubicin cardiotoxicity via inhibiting DNA-PKcs cytosolic retention and DNA damage. Acta Pharm Sin B.

[37] Pei Z, Xiong Y, Jiang S, Guo R, Jin W, Tao J, Zhang Z (2024). Heavy metal scavenger metallothionein rescues against cold stress-evoked myocardial contractile anomalies through regulation of mitophagy. Cardiovasc Toxicol.

[38] Liu Y, Yang X, Gan J, Chen S, Xiao ZX, Cao Y (2022). CB-Dock2: improved protein–ligand blind docking by integrating cavity detection, docking and homologous template fitting. Nucleic Acids Res.

[39] Yu W, Qin X, Zhang Y, Qiu P, Wang L, Zha W, Ren J (2020). Curcumin suppresses doxorubicin-induced cardiomyocyte pyroptosis via a PI3K/Akt/mTOR-dependent manner. Cardiovasc Diagn Ther.

[40] Bein K, Leikauf GD (2011). Acrolein—a pulmonary hazard. Mol Nutr Food Res.

[41] Barresi V, Trovato‐Salinaro A, Spampinato G, Musso N, Castorina S, Rizzarelli E, Condorelli DF (2016). Transcriptome analysis of copper homeostasis genes reveals coordinated upregulation of
*SLC31A1*,
*SCO1*, and
*COX11* in colorectal cancer. FEBS Open Bio.

[42] Hong SW, Page R, Truman P (2025). Smoking, coffee intake, and Parkinson’s disease: potential protective mechanisms and components. NeuroToxicology.

[43] Toda N, Okamura T (2016). Cigarette smoking impairs nitric oxide-mediated cerebral blood flow increase: implications for Alzheimer’s disease. J Pharmacol Sci.

[44] Ren J, Wold LE (2001). Measurement of cardiac mechanical function in isolated ventricular myocytes from rats and mice by computerized video-based imaging. Biol Proced Online.

[45] Wold LE, Ren J. Mechanical measurement of contractile function of isolated ventricular myocytes.
*
Methods Mol Med
* 2007, 139: 263–270. https://doi.org/10.1007/978-1-59745-571-8_17.

[46] Pei Z, Zhuang Z, Sang H, Wu Z, Meng R, He EY, Scott GI (2014). α,β-Unsaturated aldehyde crotonaldehyde triggers cardiomyocyte contractile dysfunction: role of TRPV1 and mitochondrial function. Pharmacol Res.

[47] Gao J, Liu X, Wang M, Zeng X, Wang Z, Wang Y, Lou J (2024). Adenosine protects cardiomyocytes against acrolein-induced cardiotoxicity by enhancing mitochondrial homeostasis, antioxidant defense, and autophagic flux via ERK-activated FoxO1 upregulation. Ecotoxicol Environ Saf.

[48] Ren J, Pulakat L, Whaley-Connell A, Sowers JR (2010). Mitochondrial biogenesis in the metabolic syndrome and cardiovascular disease. J Mol Med.

[49] Xu H, Cui S, Zhang Y, Ren J (2020). Mitochondrial Ca2+ regulation in the etiology of heart failure: physiological and pathophysiological implications. Acta Pharmacol Sin.

[50] Li Y, Wang X (2025). A metabolic perspective on cuproptosis. Trends Endocrinol Metab.

[51] Li C, Liu R, Xiong Z, Bao X, Liang S, Zeng H, Jin W (2024). Ferroptosis: a potential target for the treatment of atherosclerosis. Acta Biochim Biophys Sin.

[52] Zhang Y, Tu J, Li Y, Wang Y, Lu L, Wu C, Yu X (2023). Inflammation macrophages contribute to cardiac homeostasis. Cardiol Plus.

